# The enemy within: Targeting host–parasite interaction for antileishmanial drug discovery

**DOI:** 10.1371/journal.pntd.0005480

**Published:** 2017-06-08

**Authors:** Suzanne Lamotte, Gerald F. Späth, Najma Rachidi, Eric Prina

**Affiliations:** Institut Pasteur and INSERM U1201, Unité de Parasitologie Moléculaire et Signalisation, Paris, France; Northeastern University, UNITED STATES

## Abstract

The state of antileishmanial chemotherapy is strongly compromised by the emergence of drug-resistant *Leishmania*. The evolution of drug-resistant phenotypes has been linked to the parasites’ intrinsic genome instability, with frequent gene and chromosome amplifications causing fitness gains that are directly selected by environmental factors, including the presence of antileishmanial drugs. Thus, even though the unique eukaryotic biology of *Leishmania* and its dependence on parasite-specific virulence factors provide valid opportunities for chemotherapeutical intervention, all strategies that target the parasite in a direct fashion are likely prone to select for resistance. Here, we review the current state of antileishmanial chemotherapy and discuss the limitations of ongoing drug discovery efforts. We finally propose new strategies that target *Leishmania* viability indirectly via mechanisms of host–parasite interaction, including parasite-released ectokinases and host epigenetic regulation, which modulate host cell signaling and transcriptional regulation, respectively, to establish permissive conditions for intracellular *Leishmania* survival.

## Introduction

Leishmaniases are neglected diseases that prevail in tropical and subtropical areas. A recent WHO report indicates 399 million people in 11 high-burden countries and 556 million people in 12 high-burden countries are at risk for cutaneous leishmaniasis (CL) and visceral leishmaniasis (VL), respectively [1]. The incidence of human leishmaniases shows an important increase over the last decades due to multiple factors, including failing preventive and therapeutic measures, human migration caused by conflicts and political instability, global warming, and the emergence of drug-resistant parasites in developing countries [2–5]. Causal agents of leishmaniases are protozoan parasites of the *Leishmania* genus belonging to the Trypanosomatidae family. During the parasite’s life cycle, the promastigote form is transmitted by blood-feeding sandflies to vertebrate hosts, where they develop into the disease-causing amastigote form inside host phagocytes.

**Fig 1:**
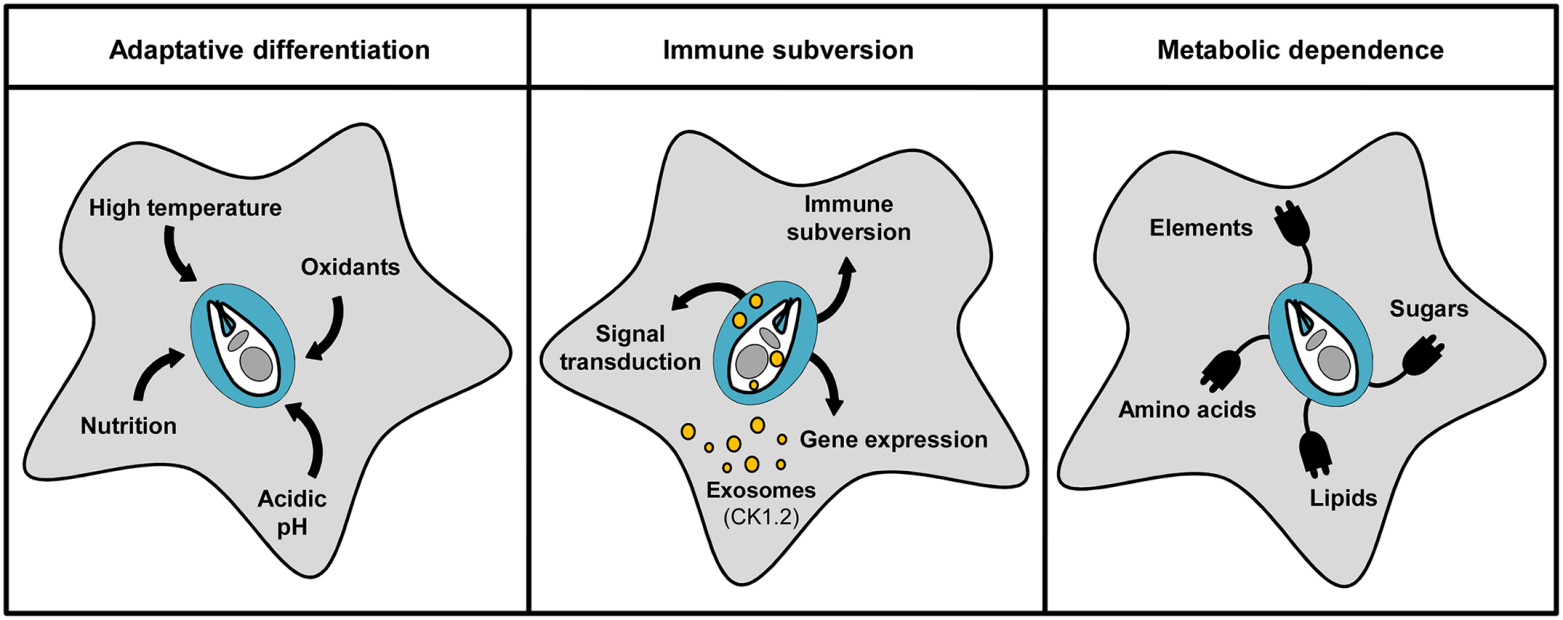
Different aspects of macrophage–*Leishmania* interaction. *Leishmania* responds to the intramacrophagic environment by adaptive differentiation (left panel) and hijacks vital macrophage functions via release of parasite ectoproteins (such as the ectokinase casein kinase 1 isoform 2 [CK1.2]), which affect host defense mechanisms, causing immune subversion (middle panel), and modulate host metabolic pathways, promoting parasite growth (right panel).

Control of intracellular *Leishmania* development relies primarily on chemotherapy but also on the ability of the parasitized host to mount an efficient immune response. The macrophage plays a key role in antiparasitic resistance but also immuno-pathology. These sentinel cells participate directly in the containment and clearance of *Leishmania* through their innate immune functions and stimulation of a protective Th1 response [6, 7]. Intracellular *Leishmania* and their host cells have coevolved intricate and dynamic interactions (Fig 1). In particular, *Leishmania* has evolved mechanisms to subvert both innate and adaptive immune responses that cause immune dysregulation and the pathologies characteristic of CL and VL and ultimately allow parasite proliferation and persistent infection inside the mammalian host [8–10]. Surprisingly, even though it is very well established that *Leishmania* reprograms its host cell to subvert the immune response and to meet the nutritional and metabolic needs for intracellular parasite survival and proliferation [11, 12], there is only little effort to exploit these crucial effects of the parasite on the host cell for antiparasitic drug discovery. Here, we review the current literature on antileishmanial therapy and *Leishmania* host–pathogen interaction and discuss novel strategies to target host cell rather than parasite biology for drug discovery—a strategy that likely will be more refractory to the emergence of drug-resistant parasites.

## Limitations of new and emerging therapies

Recent strategies to replace antimonials as first-line treatment to circumvent their limitations with respect to toxicity [13] and drug resistance [14] largely rely on repurposing of existing drugs [15]. These include the antifungal drug amphotericin B, the off-patent antibiotic paromomycin, the oral anticancer drug miltefosine, and the antimalarial drug sitamaquine, all of which were shown efficient for treating leishmaniases. Despite the success of this repurposing strategy, all these therapies have important limitations: (1) miltefosine is teratogenic, can provoke acute gastrointestinal side effects, and the length of the treatment (several weeks) causes poor treatment compliance with the risk of relapse [16], (2) conventional amphotericin B deoxycholate is not only nephrotoxic but also costly and cannot be stored at high temperature, rendering it unaffordable in some countries [17], and (3) paromomycin needs long parenteral regimens, involving qualified personnel and hospitalization [18].

In addition, depending on *Leishmania* species and geographical area, the parasite response to the drugs can vary substantially, with, for example, a cure rate of paromomycin treatment for VL ranging from 14.3% to 93.1% in Sudanese and Ethiopian patients, respectively [19]. Relapse can occur, and post-kala-azar dermal leishmaniasis can appear even months after the end of therapy [20]. These drawbacks, together with the high attrition rate observed in the leishmaniases drug discovery pipeline, caused a recent shift from the discovery of new drugs to the use of combination therapies involving 2 or more drugs at lower dosage and shorter treatment duration [21]. This is believed to overcome 2 major handicaps of current drugs, i.e., toxicity and emergence of drug-resistant parasites.

The most important limitation of current antileishmanial drugs, however, is represented by treatment failures and the emergence of drug-resistant parasites. In Bihar state (India), efficiency of antimonial therapy fell to 40% in certain hyperendemic areas [22] due to the presence of drug-resistant strains [14]. A *Leishmania infantum* strain isolated from a patient who suffered various relapses and received multiple antimonial and amphotericin B treatments was shown to be resistant to both drugs [23], suggesting that even combination therapy may be of only limited use.

## Drug target discovery exploiting *Leishmania*-specific biology

Current efforts towards antileishmanial drug discovery largely rely on the identification of target molecules that present significant structural and/or functional differences to their mammalian orthologs and are implicated in biochemical and metabolic pathways essential for parasite viability or infectivity. Following this rationale, a number of potential target candidates have been identified (reviewed in [24]) and implicated in a large variety of biological functions (S1 Table). The possibility to selectively inhibit the proteasomes of the 3 pathogenic trypanosomatids, i.e., *Leishmania* spp., *Trypanosoma cruzi*, and *T*. *brucei*, without interfering with the mammalian orthologous pathway holds great promise to discover novel treatments with broad applicability on the most important neglected tropical diseases (NTDs) [25]. Such a pan-antikinetoplastid drug may motivate Big Pharma to engage in NTD drug development, as it will increase the potential for economic return.

Despite the success of biochemical, pharmacological, and genetic approaches to validate a large number of *Leishmania* molecules as potential drug targets, any new drug that directly targets the parasite (including pan-kinetoplastid therapies) will likely have only a short therapeutic use, given the capacity of *Leishmania* to rapidly evolve towards drug-resistant phenotypes, which is partly linked to its remarkable genome plasticity. It is well established that *Leishmania* can escape drug action by modulating its gene content through various mechanisms, including gene tandem duplication, deletion, or extrachromosomal amplification, which rely on homologous recombination via interspersed repeated sequence elements [26–30]. In the absence of transcriptional regulation, *Leishmania* often resorts to chromosomal amplification as a means to modulate gene expression and to override drug pressure or adapt to a changing environment [31–34]. Likewise, mutation or deletion of transporter genes, such as those coding for aquaglyceroporine, the miltefosine transporter, or its accessory protein, LdRos3, have been linked to drug resistance [28, 35, 36].

In the following, we therefore propose host-directed therapeutic strategies as a new venue for antileishmanial drug discovery and discuss why they may be more refractory to the emergence of drug resistance.

## The impact of intracellular *Leishmania* infection on the host cell phenotype

The *Leishmania*–macrophage interaction provides an excellent example of coevolution that promotes parasite survival and causes diseases [10, 37, 38]. Conceivably, interfering with these processes represents a promising new strategy for antileishmanial intervention. In the following, we will explore the possibility to target macrophage–*Leishmania* interaction by reviewing the current literature on host cell pathways that are modulated by intracellular *Leishmania*.

### Impact on host innate and adaptive responses

Macrophages eliminate pathogenic microorganisms directly via nitric oxide (NO) or reactive oxygen species (ROS), or indirectly via the production of pro-inflammatory cytokines that initiate antimicrobial responses. *Leishmania* evades and subverts these host cell functions through the use of parasite proteins and glycolipid effectors, which are either expressed on the parasite surface or released into the cytoplasm, where they target host cell signaling processes [39–42]. Additionally, *Leishmania* interferes with the antigen-presentation capacity of their host cells through multiple mechanisms, implicating changes in abundance of costimulatory molecules or Major Histocompatibility Complex (MHC)-peptide complexes, destabilization of lipid rafts, or sequestration of *Leishmania* antigens [42–48]. Finally, *Leishmania* can interfere with the expression of microRNAs, which are considered as master regulators of the cellular transcriptome with important immunomodulatory functions (reviewed in [49]). In conclusion, a better understanding of how *Leishmania* interferes with macrophage immune functions may open important new venues to rescue the host cell’s immune potential by immunotherapy or immunochemotherapy, for example, using pro-inflammatory cytokines and chemokines alone or in combination with antileishmanial drugs (reviewed in [50, 51]).

### Impact on host cell viability

One of the striking features of the molecular dialogue between *Leishmania* and its host cell is the increased life span observed for parasite-infected macrophages. Since the seminal study of Moore and Matlashewski suggesting a *Leishmania*-dependent inhibition of host cell apoptosis [52], this observation has been confirmed by various reports [53, 54]. More recent reports studying the anti-apoptotic effect observed in the VL mouse model proposed molecular mechanisms involving *Leishmania*-CpG motifs, host myeloid cell leukemia 1 factor (MCL-1), and the cAMP response element binding protein (CREB) transcription factor [55, 56].

### Impact on host cell metabolism

Auxotrophy of the intracellular *Leishmania* amastigote developmental stage for various essential nutrients renders this parasite dependent upon host resources for its growth during mammalian colonization [38, 57]. It is therefore not surprising that the impact of *Leishmania* infection on the host cell transcriptome translates into important metabolic changes that fuel parasite intracellular growth. Indeed, various reports demonstrated the up-regulation of genes coding for key molecules involved in sterol and fatty acid metabolisms during the early phase of infection and during active multiplication of intracellular parasites [11, 12, 58]. These transcriptional studies were supported by proteomics findings demonstrating the establishment of a *Leishmania*-specific macrophage protein expression profile with singular features related to major metabolic pathways [59–61]. Together, these data confirm that *Leishmania* turn their host cells into metabolic factories to ensure intracellular amastigote growth. Conceivably, this metabolic dependence of the parasite on the host cell may open new venues to eliminate intracellular *Leishmania*, for example, by pharmacological restoration of normal macrophage metabolic functions that may cause parasite death by starvation.

## Targeting host–pathogen interaction for chemotherapeutic intervention

Targeting the host for antimicrobial therapy has been recognized as a new and fertile venue to treat viral, bacterial, and fungal diseases that provides the advantage to dramatically increase the genetic barrier for drug resistance [62–64]. Such host-directed therapies largely depend either on drugs developed for noncommunicable diseases that show good safety profiles or various forms of immunotherapeutic intervention [65, 66]. The possibility to adopt the same strategy against *Leishmania* is supported by reports on the antileishmanial effects of imiquimod, which acts as a Toll-Like Receptor (TLR) agonist [67], or the compound Naloxonazine, which kills intracellular *Leishmania* by targeting host cell vATPases [68].

In the following, we propose parasite-released ectoproteins that can affect host cell signal transduction *in trans* and host histone-modifying enzymes that may be subverted by intracellular *Leishmania* as possible targets for the discovery of host-directed drug candidates (S2 Table).

### Targeting *Leishmania* ectokinases and modulation of host cell signaling

Although the impact of intracellular *Leishmania* on host cell immune signaling and pathogenesis has been recognized [9, 43, 69], little information is available on how parasite signaling proteins, particularly released protein kinases, are involved in the modulation of host cell signaling. The importance of released kinases in the survival of intracellular parasites is well illustrated by members of the *Toxoplasma* ROP kinases family, with ROP16 phosphorylating and activating STAT3 [70, 71], thus mimicking anti-inflammatory signal transduction. Likewise, members of the *Plasmodium* FIK kinase protein family [72] that are exported into the erythrocyte cytoplasm shortly after infection were implicated in remodeling host cell membrane and cytoskeleton, with a likely impact on cytoadhesion [73]. In contrast, the role of *Leishmania* ectokinases in intracellular infection has not been studied in detail. Studies on the secretome and the exosomal proteome in *L*. *donovani* promastigotes identified over 400 putative ectoproteins [74, 75], including 13 secreted and 14 exosomal kinases, the majority of which are involved in biochemical processes (glycolytic pathways, nucleotide synthesis), suggesting an important effect on the host cell metabolism. Only 3 signaling kinases were identified, i.e., the mitogen-activated protein kinases MPK3 and MPK11 and Casein kinase 1 isoform 2 (CK1.2). Only CK1.2 has been functionally linked to infection and host cell immune subversion and, thus, is further described below.

CK1.2 (LmjF.35.1010) is a serine/threonine protein kinase that has attracted considerable interest as a putative drug target. Known CK1 inhibitors have been shown to block growth of extracellular *Leishmania* in in vitro culture [76] and intracellular parasites established in primary murine macrophages [77, 78]. *Leishmania* CK1.2 is a highly conserved kinase with over 70% of identity with its human ortholog, suggesting that the evolution of this kinase is driven by its interaction with host cell substrates. CK1.2 is also the most conserved kinase across all *Leishmania* species (over 99% of identity), further supporting the notion that its evolution may be uncoupled from species-specific constraints and driven by interaction with host proteins during intracellular infection [78]. Additionally, CK1.2 can directly phosphorylate host substrates, such as the human complement component C3a [79, 80] or the human receptor IFNAR1 attenuating the cellular responses to IFNα in vitro [81].

CK1.2 and other kinases thus likely impact on host cell signaling and metabolism to establish permissive conditions for intracellular *Leishmania* survival. Targeting such parasite-released “trans-acting” signaling factors constitutes a very interesting novel approach for the development of antileishmanials for 2 reasons: first, kinase inhibitors are prime candidates to treat various human pathologies, including cancer, diabetes, or inflammation. Thus, drug-discovery efforts directed to target *Leishmania*-released kinases can benefit from the availability of dedicated libraries that have already been well characterized. Second, it is conceivable that inhibitors that target *Leishmania*-released kinases that only affect the host may be more refractory for the development of drug resistance. Finally, mutation of these kinases may affect their role in host cell immune evasion and thus be strongly detrimental for parasite survival.

### Targeting *Leishmania*-dependent epigenetic host cell reprogramming

The important impact of intracellular pathogens on the host cell transcriptome incited studies on the epigenetic consequences of infection. Epigenetics refers to heritable changes in gene expression that do not involve modifications of the underlying DNA sequence but depend on alteration of either DNA or of histone proteins influencing chromatin structure and local gene expression. Various pathogens modulate host cell DNA methylation levels to inhibit expression of genes that are involved in clearance of the infectious agent but also increase expression of genes that promote microbial growth and survival. For example, changes in DNA methylation levels were linked to (1) attenuated NFKB1- and IRF2-mediated pro-inflammatory signaling during *Mycobacterium tuberculosis* infection [82], (2) modulation of the inflammatory response, apoptosis, and pathogen-induced signaling during *Burkholderia pseudomallei* infection [83], and (3) changes in host behavior as observed in *Toxoplasma gondii*–infected mice, in which a decrease in the methylation levels of the arginine vasopressin promoter and subsequent increased neuronal gene expression were linked to fear reversion against the natural predator, promoting parasite transmission [84]. In contrast to these examples, only very limited information is available on the epigenetic impact of *Leishmania* infection on the host cell, with only 1 recent study showing that *L*. *donovani* causes epigenetic variation in macrophage DNA methylation, thus interfering with genes implicated in host cell antimicrobial defense [85].

Infectious microbes also remodel the chromatin and its accessibility by altering histone modifications. For example, *T*. *gondii* infection blocks Histone 3 (H3) phosphorylation of serine 10 and acetylation of lysines 4 and 9 in the promoter of TNFα, thus causing a transcriptional downregulation of this pro-inflammatory cytokine [86]. Likewise, decreased histone acetylation during *T*. *gondii* infection has been linked to altered STAT1 binding to INFγ-regulated promoters, which was reversed by treatment with histone deacetylase inhibitors [87]. *Theileria-*induced SMYD3 methyltransferase activity increases histone 3 lysine 4 trimethylation in the promoter of the host cell matrix metalloproteinase 9 gene, and increased expression of this protein has been linked to the invasive phenotype of infected cells [88].

In addition, secreted microbial proteins have been shown to interfere with host epigenetic control and gene expression. Influenza virus NS1 shares similarity with the H3 tail that binds the human polymerase-associated factor 1 complex, thus attenuating antiviral gene expression [89]. During *Listeria monocytogenes* infection, the host deacetylase sirtuin 2 (SIRT2) translocates to the nucleus, causing deacetylation of H3K18, thereby facilitating infection by repressing a specific set of genes [90]. *Chlamydia trachomatis* and *Legionella pneumophila* use a similar mechanism, secreting proteins with a conserved SET domain that specifically methylates host cell histones, allowing direct regulation of host gene expression [91, 92].

Surprisingly, despite the massive effect of *Leishmania* infection on the host cell transcriptome and the potential effect of parasite-released proteases on the nuclear proteome [93], no information is available on how the parasite affects histone modification of the host macrophage. The *Leishmania* genome encodes for an important number of putative histone-modifying enzymes (HMEs) [94]. In light of the largely constitutive gene expression in these early-branching eukaryotes, it is interesting to speculate that some of these proteins may be released and modify host cell histones to establish permissive conditions for intracellular survival. Alternatively, *Leishmania* infection may alter the activity of host cell HMEs that establishes an epigenetic profile permissive for intracellular parasite survival. Targeting parasite-released HMEs or directly modulating the host cell epigenome opens exciting new avenues for antileishmanial therapies that may be more refractory to the emergence of drug resistance. This possibility is supported by recent findings demonstrating that the antileishmanial effect of imipramine, an antidepressant, is mediated via its effect on host cell HDAC11, which decreases IL10 expression [95], thus overcoming a host cell-dependent mechanism of antimony resistance.

## Conclusions

**Fig 2:**
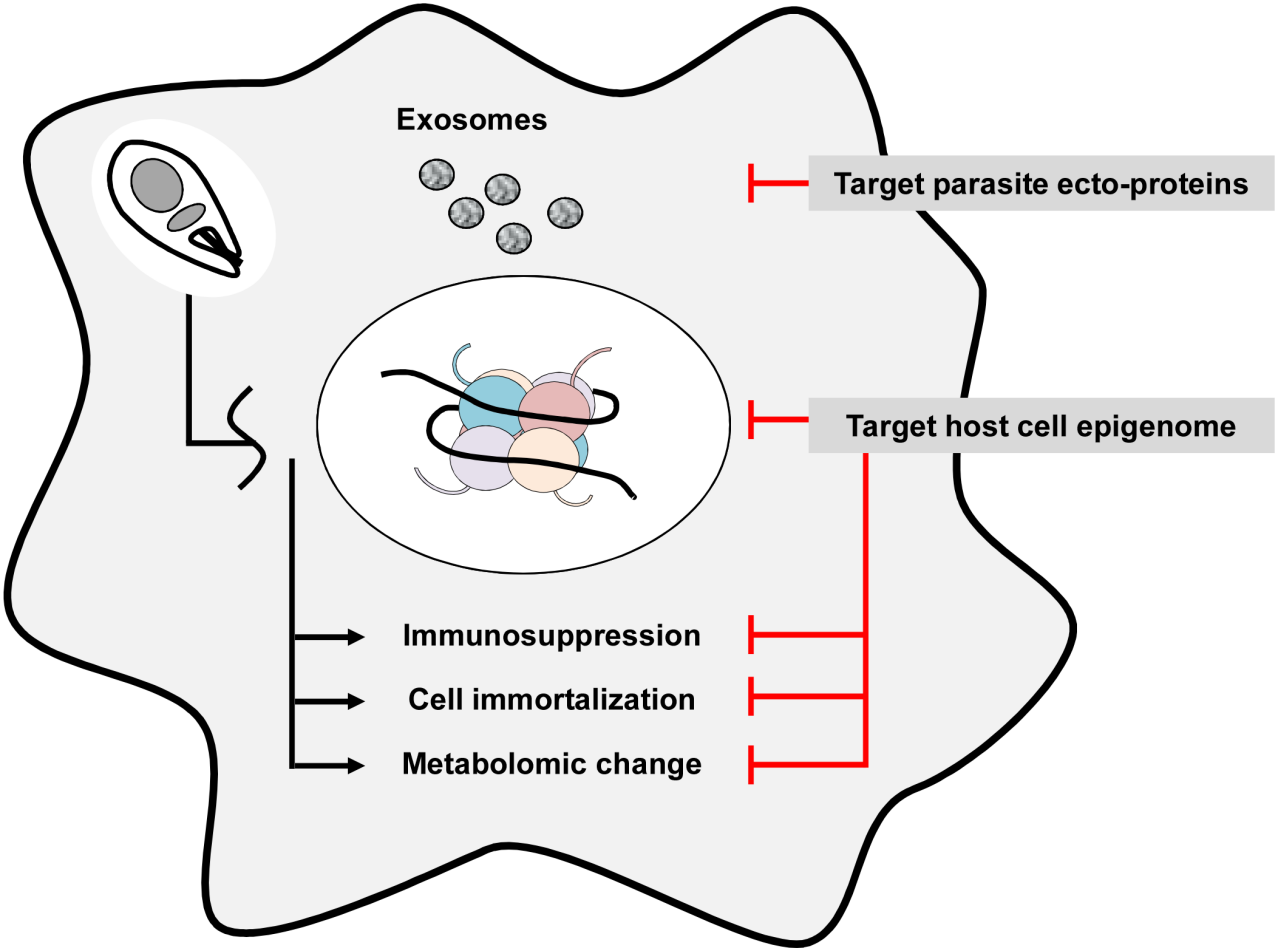
Targeting host–parasite interaction as a new venue for antileishmanial drug discovery. Exosomal or secreted parasite factors released into the host cell likely modulate the macrophage epigenome, causing phenotypic changes that favor parasite survival, including suppression of immune functions, prolongation of host cell survival, and metabolic changes necessary for parasite proliferation. Interfering with parasite factors that act *in trans* on the host cell or restoration of the normal host cell epigenome will likely interfere with intracellular parasite survival and may thus be exploited for antileishmanial drug discovery.

In conclusion, the capacity of *Leishmania* to evolve towards a drug-resistant phenotype calls for new concepts for antileishmanial drug discovery. Our review proposes such new strategies by (1) targeting parasite ectokinases that modulate the host cell phenotype to establish permissive conditions for parasite survival, and (2) targeting host cell histone-modifying enzymes to restore a normal macrophage transcript profile that may be deleterious for intracellular *Leishmania* survival (Fig 2). Systems-level approaches combining high-throughput sequencing, proteomics and metabolomics analyses, RNAi screening, and pharmacological assessment need to be conducted to establish the proof-of-principle for these strategies and discover and validate such novel targets. Drug targets that are not under the direct genetic control of the parasite may allow the discovery of inhibitors that are likely more refractory to classical mechanisms of drug resistance that often involves mutation of target gene or uptake systems, or amplification of efflux pumps.

Despite their great promise, host-directed therapies bear their own new challenges. First, given the differences in lifestyle and pathogenicity between the 3 major trypanosomatid pathogens, with *T*. *brucei* being an obligate extracellular pathogen and *T*. *cruzi* infecting various mammalian cells, the development of a pan-kinetoplastid therapy seems difficult. This fact is further illustrated by a recent high-throughput drug-screening campaign against all 3 trypanosomatids that identified only a few compounds with broad activity [96]. Even though one may expect that this will turn down Big Pharma from host-directed therapies, quite on the contrary, this strategy may actually incite important interest, as macrophages are the host cells of various viral, bacterial, and fungal pathogens with major global public health impact. It is conceivable that these pathogens have evolved intracellular survival strategies that are analogous to the ones employed by *Leishmania*, opening the exciting and yet unexplored possibility of pan-intracellular pathogen therapies.

A second major concern for host-directed therapies is toxicity. However, many host functions are the targets of successful therapies against various noncommunicable diseases, such as cancer or autoimmunity, and a repurposing strategy is already successfully applied on various infectious diseases. For example, protein kinases and epigenetic enzymes represent some of the most important groups of drug targets currently in development for various human diseases and are the subject of several U.S. Food and Drug Administration (FDA)–approved drugs, opening the interesting venue to repurpose existing treatments with good safety profiles for antileishmanial chemotherapy.

**Key learning points**

- High attrition rate in drug discovery pipeline for leishmaniases means very few new candidate drugs available.
- *Leishmania* genome plasticity allows swift adaptation to new antiparasitic drugs.
- Targeting *Leishmania*–host cell interactions is a novel strategy to circumvent *Leishmania* drug resistance.
- *Leishmania* ectoproteins are likely more refractory to drug resistance.
- Modulation of host cell epigenome is important to control intracellular *Leishmania* growth.

**Top five papers**

1. Nagle AS, Khare S, Kumar AB, Supek F, Buchynskyy A, Mathison CJ, et al. Recent developments in drug discovery for leishmaniasis and human African trypanosomiasis. Chem Rev. 2014 Nov 26;114(22):11305-47.doi 10.1021/cr500365f. http://www.ncbi.nlm.nih.gov/pubmed/25365529 PMID:25365529
2. Peña I, Pilar Manzano M, Cantizani J, Kessler A, Alonso-Padilla J, Bardera AI, et al. New compound sets identified from high throughput phenotypic screening against three kinetoplastid parasites: an open resource. Sci Rep. 2015;5:8771.doi: 10.1038/srep08771. http://www.ncbi.nlm.nih.gov/pubmed/25740547
3. Khare S, Nagle AS, Biggart A, Lai YH, Liang F, Davis LC, et al. Proteasome inhibition for treatment of leishmaniasis, Chagas disease and sleeping sickness. Nature. 2016 Aug 8.doi: 10.1038/nature19339. http://www.ncbi.nlm.nih.gov/pubmed/27501246
4. Leprohon P, Fernandez-Prada C, Gazanion E, Monte-Neto R, Ouellette M. Drug resistance analysis by next generation sequencing in Leishmania. Int J Parasitol Drugs Drug Resist. 2015 Apr;5(1):26-35.doi: 10.1016/j.ijpddr.2014.09.005. http://www.ncbi.nlm.nih.gov/pubmed/25941624
5. Marr AK, MacIsaac JL, Jiang R, Airo AM, Kobor MS, McMaster WR. Leishmania donovani infection causes distinct epigenetic DNA methylation changes in host macrophages. PLoS Pathog. 2014 Oct;10(10):e1004419.doi: 10.1371/journal.ppat.1004419. http://www.ncbi.nlm.nih.gov/pubmed/25299267

## Supporting information

S1 Table Potential drug targets expressed by *Leishmania*. (PDF)

S2 Table Potential drug targets expressed by the host cell or secreted by *Leishmania*. (PDF)
