# Supplementary material for: The enemy within: Targeting host–parasite interaction for antileishmanial drug discovery
Source: PLoS Negl Trop Dis. 2017 Jun 8;11(6):e0005480. doi: 10.1371/journal.pntd.0005480 (PMC5464532; doi:10.1371/journal.pntd.0005480)
Supplement: S1 Table — (PDF) [file pntd.0005480.s001.pdf]

Sup Table 1. Potential drug targets expressed by *Leishmania*

Category	Pathways / enzymes	Reference
Detoxification	trypanothione pathway, glyoxalase system, peroxidase and superoxide dismutase	(1-4)
Nucleotide & Lipid metabolism	polyamine, folate and sterol metabolisms	(5-9)
Energy metabolism	glycosomal and mitochondrial enzymes	(10-12)
Post-translational modification & signaling	enzymes involved in ubiquitination, myristoylation, phosphorylation processes, calcium homeostasis	(13-17)
Proteolysis and turnover	metallo-proteases, serine and cysteine proteases, metacaspases, proteasomal proteins	(18, 19)
Transporter molecules	for nucleotides, fatty acids, metals, amino acids and polyamines	(20-24)
Topoisomerases	type II	(25)
Cell cycle regulation/parasite stage differentiation	heat-shock proteins, kinases	(26-31)
Unknown parasite-specific functions	hypothetical proteins, proteins of prokaryotic origin	(32-37)

Reference list

1. Benitez D, Medeiros A, Fiestas L, Panozzo-Zenere EA, Maiwald F, Prousis KC, et al. Identification of Novel Chemical Scaffolds Inhibiting Trypanothione Synthetase from Pathogenic Trypanosomatids. PLoS Negl Trop Dis. 2016 Apr;10(4):e0004617. doi: 10.1371/journal.pntd.0004617. http://www.ncbi.nlm.nih.gov/pubmed/27070550. PMID:27070550

2. Leroux AE, Krauth-Siegel RL. Thiol redox biology of trypanosomatids and potential targets for chemotherapy. Mol Biochem Parasitol. 2016 Mar-Apr;206(1-2):67-74. doi: 10.1016/j.molbiopara.2015.11.003. http://www.ncbi.nlm.nih.gov/pubmed/26592324. PMID:26592324

3. Sousa Silva M, Ferreira AE, Tomas AM, Cordeiro C, Ponces Freire A. Quantitative assessment of the glyoxalase pathway in *Leishmania infantum* as a therapeutic target by modelling and computer simulation. Febs J. 2005 May;272(10):2388-98. doi: 10.1111/j.1742-4658.2005.04632.x. http://www.ncbi.nlm.nih.gov/pubmed/15885089. PMID:15885089

4. Padmanabhan PK, Mukherjee A, Singh S, Chattopadhyaya S, Gowri VS, Myler PJ, et al. Glyoxalase I from *Leishmania donovani*: a potential target for anti-parasite drug. Biochem Biophys Res Commun. 2005 Dec 2;337(4):1237-48. doi: 10.1016/j.bbrc.2005.09.179. http://www.ncbi.nlm.nih.gov/pubmed/16236261. PMID:16236261

5. Ilari A, Fiorillo A, Baiocco P, Poser E, Angiulli G, Colotti G. Targeting polyamine metabolism for finding new drugs against leishmaniasis: a review. Mini Rev Med Chem. 2015;15(3):243-52. http://www.ncbi.nlm.nih.gov/pubmed/25769972. PMID:25769972

6. de Macedo-Silva ST, Visbal G, Urbina JA, de Souza W, Rodrigues JC. Potent In Vitro Antiproliferative Synergism of Combinations of Ergosterol Biosynthesis Inhibitors against *Leishmania amazonensis*. Antimicrob Agents Chemother. 2015 Oct;59(10):6402-18. doi: 10.1128/AAC.01150-15. http://www.ncbi.nlm.nih.gov/pubmed/26239973. PMID:26239973

7. Pomel S, Cojean S, Loiseau PM. Targeting sterol metabolism for the development of antileishmanials. Trends Parasitol. 2015 Jan;31(1):5-7. doi: 10.1016/j.pt.2014.11.007. http://www.ncbi.nlm.nih.gov/pubmed/25498194. PMID:25498194

8. Roberts CW, McLeod R, Rice DW, Ginger M, Chance ML, Goad LJ. Fatty acid and sterol metabolism: potential antimicrobial targets in apicomplexan and trypanosomatid parasitic protozoa. Mol Biochem Parasitol. 2003 Feb;126(2):129-42. http://www.ncbi.nlm.nih.gov/pubmed/12615312. PMID:12615312

9. Nare B, Luba J, Hardy LW, Beverley S. New approaches to *Leishmania* chemotherapy: pteridine reductase 1 (PTR1) as a target and modulator of antifolate sensitivity. Parasitology. 1997;114 Suppl:S101-10. http://www.ncbi.nlm.nih.gov/pubmed/9309772. PMID:9309772

10. Moyersoen J, Choe J, Fan E, Hol WG, Michels PA. Biogenesis of peroxisomes and glycosomes: trypanosomatid glycosome assembly is a promising new drug target. FEMS Microbiol Rev. 2004 Nov;28(5):603-43. doi: 10.1016/j.femsre.2004.06.004. http://www.ncbi.nlm.nih.gov/pubmed/15539076. PMID:15539076
11. Silva LA, Vinaud MC, Castro AM, Cravo PV, Bezerra JC. In silico search of energy metabolism inhibitors for alternative leishmaniasis treatments. Biomed Res Int. 2015;2015:965725. doi: 10.1155/2015/965725. http://www.ncbi.nlm.nih.gov/pubmed/25918726. PMID:25918726
12. Ortiz D, Forquer I, Boitz J, Soysa R, Elya C, Fulwiler A, et al. Targeting the Cytochrome bc1 Complex of *Leishmania* Parasites for Discovery of Novel Drugs. Antimicrob Agents Chemother. 2016 Aug;60(8):4972-82. doi: 10.1128/AAC.00850-16. http://www.ncbi.nlm.nih.gov/pubmed/27297476. PMID:27297476
13. Gannavaram S, Bhattacharya P, Ismail N, Kaul A, Singh R, Nakhasi HL. Modulation of Innate Immune Mechanisms to Enhance *Leishmania* Vaccine-Induced Immunity: Role of Coinhibitory Molecules. Front Immunol. 2016;7:187. doi: 10.3389/fimmu.2016.00187. http://www.ncbi.nlm.nih.gov/pubmed/27242794. PMID:27242794
14. Wright MH, Paape D, Storck EM, Serwa RA, Smith DF, Tate EW. Global analysis of protein N-myristoylation and exploration of N-myristoyltransferase as a drug target in the neglected human pathogen *Leishmania donovani*. Chem Biol. 2015 Mar 19;22(3):342-54. doi: 10.1016/j.chembiol.2015.01.003. http://www.ncbi.nlm.nih.gov/pubmed/25728269. PMID:25728269
15. Brannigan JA, Roberts SM, Bell AS, Hutton JA, Hodgkinson MR, Tate EW, et al. Diverse modes of binding in structures of *Leishmania major* N-myristoyltransferase with selective inhibitors. IUCrJ. 2014 Jul 1;1(Pt 4):250-60. doi: 10.1107/S2052252514013001. http://www.ncbi.nlm.nih.gov/pubmed/25075346. PMID:25075346
16. Nascimento M, Zhang WW, Ghosh A, Houston DR, Berghuis AM, Olivier M, et al. Identification and characterization of a protein-tyrosine phosphatase in *Leishmania*: Involvement in virulence. J Biol Chem. 2006 Nov 24;281(47):36257-68. doi: 10.1074/jbc.M606256200. http://www.ncbi.nlm.nih.gov/pubmed/17005559. PMID:17005559
17. Benaim B, Garcia CR. Targeting calcium homeostasis as the therapy of Chagas' disease and leishmaniasis - a review. Trop Biomed. 2011 Dec;28(3):471-81. http://www.ncbi.nlm.nih.gov/pubmed/22433874. 22433874
18. Singh J, Srivastava A, Jha P, Sinha KK, Kundu B. L-Asparaginase as a new molecular target against leishmaniasis: insights into the mechanism of action and structure-based inhibitor design. Mol Biosyst. 2015 Jul;11(7):1887-96. doi: 10.1039/c5mb00251f. http://www.ncbi.nlm.nih.gov/pubmed/25893895. PMID:25893895
19. Das P, Alam MN, Paik D, Karmakar K, De T, Chakraborti T. Protease inhibitors in potential drug development for Leishmaniasis. Indian J Biochem Biophys. 2013 Oct;50(5):363-76. http://www.ncbi.nlm.nih.gov/pubmed/24772958. PMID:24772958
20. de Koning HP, Bridges DJ, Burchmore RJ. Purine and pyrimidine transport in pathogenic protozoa: from biology to therapy. FEMS Microbiol Rev. 2005 Nov;29(5):987-1020. doi: 10.1016/j.femsre.2005.03.004. http://www.ncbi.nlm.nih.gov/pubmed/16040150. PMID:16040150
21. Landfear SM, Ullman B, Carter NS, Sanchez MA. Nucleoside and nucleobase transporters in parasitic protozoa. Eukaryot Cell. 2004 Apr;3(2):245-54. http://www.ncbi.nlm.nih.gov/pubmed/15075255. PMID:15075255
22. Huynh C, Sacks DL, Andrews NW. A *Leishmania amazonensis* ZIP family iron transporter is essential for parasite replication within macrophage phagolysosomes. J Exp Med. 2006 Oct 2;203(10):2363-75. doi: 10.1084/jem.20060559. http://www.ncbi.nlm.nih.gov/pubmed/17000865. PMID:17000865
23. Alberca LN, Sbaraglini ML, Balcazar D, Fraccaroli L, Carrillo C, Medeiros A, et al. Discovery of novel polyamine analogs with anti-protozoal activity by computer guided drug repositioning. J Comput Aided Mol Des. 2016 Apr;30(4):305-21. doi: 10.1007/s10822-016-9903-6. http://www.ncbi.nlm.nih.gov/pubmed/26891837. PMID:26891837
24. Mittra B, Laranjeira-Silva MF, Perrone Bezerra de Menezes J, Jensen J, Michailowsky V, Andrews NW. A Trypanosomatid Iron Transporter that Regulates Mitochondrial Function Is Required for *Leishmania amazonensis* Virulence. PLoS Pathog. 2016 Jan;12(1):e1005340. doi: 10.1371/journal.ppat.1005340. http://www.ncbi.nlm.nih.gov/pubmed/26741360. PMID:26741360
25. Singh G, Dey CS. Induction of apoptosis-like cell death by pentamidine and doxorubicin through differential inhibition of topoisomerase II in arsenite-resistant *L. donovani*. Acta Trop. 2007 Sep;103(3):172-85. doi: 10.1016/j.actatropica.2007.06.004. http://www.ncbi.nlm.nih.gov/pubmed/17655815. PMID:17655815
26. Requena JM, Montalvo AM, Fraga J. Molecular Chaperones of *Leishmania*: Central Players in Many Stress-Related and -Unrelated Physiological Processes. Biomed Res Int. 2015;2015:301326. doi: 10.1155/2015/301326. http://www.ncbi.nlm.nih.gov/pubmed/26167482. PMID:26167482

27. Doerig C, Späth G, Wiese M. Protein phosphorylation in parasites. Selzer PM, editor: WILEY Blackwell; 2014.
28. Merritt C, Silva LE, Tanner AL, Stuart K, Pollastri MP. Kinases as druggable targets in trypanosomatid protozoan parasites. Chem Rev. 2014 Nov 26;114(22):11280-304. doi: 10.1021/cr500197d. http://www.ncbi.nlm.nih.gov/pubmed/26443079. PMID:26443079
29. Dacher M, Morales MA, Pescher P, Leclercq O, Rachidi N, Prina E, et al. Probing druggability and biological function of essential proteins in *Leishmania* combining facilitated null mutant and plasmid shuffle analyses. Mol Microbiol. 2014 Jul;93(1):146-66. doi: 10.1111/mmi.12648. http://www.ncbi.nlm.nih.gov/pubmed/24823804. PMID:24823804
30. Naula C, Parsons M, Mottram JC. Protein kinases as drug targets in trypanosomes and *Leishmania*. Biochim Biophys Acta. 2005 Dec 30;1754(1-2):151-9. doi: 10.1016/j.bbapap.2005.08.018. http://www.ncbi.nlm.nih.gov/pubmed/16198642. PMID:16198642
31. Brumlik MJ, Pandeswara S, Ludwig SM, Murthy K, Curiel TJ. Parasite mitogen-activated protein kinases as drug discovery targets to treat human protozoan pathogens. J Signal Transduct. 2011;2011:971968. doi: 10.1155/2011/971968. http://www.ncbi.nlm.nih.gov/pubmed/21637385. PMID:21637385
32. Kumar SS, Gokulasuriyan RK, Ghosh M. Comparative *in silico* genome analysis of *Leishmania* (*Leishmania*) *donovani*: A step towards its species specificity. Meta Gene. 2014 Dec;2:782-98. doi: 10.1016/j.mgene.2014.10.003. http://www.ncbi.nlm.nih.gov/pubmed/25606461. PMID:25606461
33. Nirujogi RS, Pawar H, Renuse S, Kumar P, Chavan S, Sathe G, et al. Moving from unsequenced to sequenced genome: reanalysis of the proteome of *Leishmania donovani*. J Proteomics. 2014 Jan 31;97:48-61. doi: 10.1016/j.jprot.2013.04.021. http://www.ncbi.nlm.nih.gov/pubmed/23665000. PMID:23665000
34. Leprohon P, Fernandez-Prada C, Gazanion E, Monte-Neto R, Ouellette M. Drug resistance analysis by next generation sequencing in *Leishmania*. Int J Parasitol Drugs Drug Resist. 2015 Apr;5(1):26-35. doi: 10.1016/j.ijpddr.2014.09.005. http://www.ncbi.nlm.nih.gov/pubmed/25941624. PMID:25941624
35. Berg M, Mannaert A, Vanaerschot M, Van Der Auwera G, Dujardin JC. (Post-) Genomic approaches to tackle drug resistance in *Leishmania*. Parasitology. 2013 Oct;140(12):1492-505. doi: 10.1017/S0031182013000140. http://www.ncbi.nlm.nih.gov/pubmed/23480865. PMID:23480865
36. Moreira Dde S, Pescher P, Laurent C, Lenormand P, Spath GF, Murta SM. Phosphoproteomic analysis of wild-type and antimony-resistant *Leishmania braziliensis* lines by 2D-DIGE technology. Proteomics. 2015 Sep;15(17):2999-3019. doi: 10.1002/pmic.201400611. http://www.ncbi.nlm.nih.gov/pubmed/25959087. PMID:25959087
37. Vikeved E, Backlund A, Alsmark C. The Dynamics of Lateral Gene Transfer in Genus *Leishmania* - A Route for Adaptation and Species Diversification. PLoS Negl Trop Dis. 2016 Jan;10(1):e0004326. doi: 10.1371/journal.pntd.0004326. http://www.ncbi.nlm.nih.gov/pubmed/26730948. PMID:26730948
