# Supplementary material for: The enemy within: Targeting host–parasite interaction for antileishmanial drug discovery
Source: PLoS Negl Trop Dis. 2017 Jun 8;11(6):e0005480. doi: 10.1371/journal.pntd.0005480 (PMC5464532; doi:10.1371/journal.pntd.0005480)
Supplement: S2 Table — (PDF) [file pntd.0005480.s002.pdf]

Sup Table 2. Potential drug targets expressed by the host cell or secreted by *Leishmania.*

Category	Molecular effector	Reference
Host immune response	retinoid X receptor alpha, deubiquitinating enzyme A20, CTLA-4, Programed Death-1, CD200R, TIM-3, miR122 or Dicer molecules, pro-inflammatory cytokines and chemokines	(1-6)
Host cell viability	host myeloid cell leukemia 1 factor shRNA	(7)
Host cell metabolism	sterol, cholesterol, carbohydrate	(8-10)
Epigenetic host cell reprogramming	DNA demethylase, HMEs	(11, 12)
Leishmania ectokinases	CK1.2	(13-15)

Reference list

1. Gannavaram S, Bhattacharya P, Ismail N, Kaul A, Singh R, Nakhasi HL. Modulation of Innate Immune Mechanisms to Enhance *Leishmania* Vaccine-Induced Immunity: Role of Coinhibitory Molecules. Front Immunol. 2016;7:187. doi: 10.3389/fimmu.2016.00187. http://www.ncbi.nlm.nih.gov/pubmed/27242794. PMID:27242794
2. Singh OP, Sundar S. Immunotherapy and targeted therapies in treatment of visceral leishmaniasis: current status and future prospects. Front Immunol. 2014;5:296. doi: 10.3389/fimmu.2014.00296. http://www.ncbi.nlm.nih.gov/pubmed/25183962. PMID:25183962
3. Roatt BM, Aguiar-Soares RD, Coura-Vital W, Ker HG, Moreira N, Vitoriano-Souza J, et al. Immunotherapy and Immunochemotherapy in Visceral Leishmaniasis: Promising Treatments for this Neglected Disease. Front Immunol. 2014;5:272. doi: 10.3389/fimmu.2014.00272. http://www.ncbi.nlm.nih.gov/pubmed/24982655. PMID:24982655
4. Beattie L, d'El-Rei Hermida M, Moore JW, Maroof A, Brown N, Lagos D, et al. A transcriptomic network identified in uninfected macrophages responding to inflammation controls intracellular pathogen survival. Cell Host Microbe. 2013 Sep 11;14(3):357-68. doi: 10.1016/j.chom.2013.08.004. http://www.ncbi.nlm.nih.gov/pubmed/24034621. PMID:24034621
5. Srivastav S, Kar S, Chande AG, Mukhopadhyaya R, Das PK. *Leishmania donovani* exploits host deubiquitinating enzyme A20, a negative regulator of TLR signaling, to subvert host immune response. J Immunol. 2012 Jul 15;189(2):924-34. doi: 10.4049/jimmunol.1102845. http://www.ncbi.nlm.nih.gov/pubmed/22685311. PMID:22685311
6. Ghosh J, Bose M, Roy S, Bhattacharyya SN. *Leishmania donovani* targets Dicer1 to downregulate miR-122, lower serum cholesterol, and facilitate murine liver infection. Cell Host Microbe. 2013 Mar 13;13(3):277-88. doi: 10.1016/j.chom.2013.02.005. http://www.ncbi.nlm.nih.gov/pubmed/23498953. PMID:23498953
7. Giri J, Srivastav S, Basu M, Palit S, Gupta P, Ukil A. *Leishmania donovani* Exploits Myeloid Cell Leukemia 1 (MCL-1) Protein to Prevent Mitochondria-dependent Host Cell Apoptosis. J Biol Chem. 2016 Feb 12;291(7):3496-507. doi: 10.1074/jbc.M115.672873. http://www.ncbi.nlm.nih.gov/pubmed/26670606. PMID:26670606
8. Rabhi I, Rabhi S, Ben-Othman R, Rasche A, Daskalaki A, Trentin B, et al. Transcriptomic signature of *Leishmania* infected mice macrophages: a metabolic point of view. PLoS Negl Trop Dis. 2012;6(8):e1763. doi: 10.1371/journal.pntd.0001763. http://www.ncbi.nlm.nih.gov/pubmed/22928052. PMID:22928052
9. Osorio y Fortea J, de La Llave E, Regnault B, Coppee JY, Milon G, Lang T, et al. Transcriptional signatures of BALB/c mouse macrophages housing multiplying *Leishmania amazonensis* amastigotes. BMC Genomics. 2009;10:119. doi: 10.1186/1471-2164-10-119. http://www.ncbi.nlm.nih.gov/pubmed/19302708. PMID:19302708
10. Dillon LA, Suresh R, Okrah K, Corrada Bravo H, Mosser DM, El-Sayed NM. Simultaneous transcriptional profiling of *Leishmania major* and its murine macrophage host cell reveals insights into host-pathogen interactions. BMC Genomics. 2015;16(1):1108. doi: 10.1186/s12864-015-2237-2. http://www.ncbi.nlm.nih.gov/pubmed/26715493. PMID:26715493
11. Marr AK, MacIsaac JL, Jiang R, Airo AM, Kobor MS, McMaster WR. *Leishmania donovani* infection causes distinct epigenetic DNA methylation changes in host macrophages. PLoS Pathog. 2014 Oct;10(10):e1004419. doi: 10.1371/journal.ppat.1004419. http://www.ncbi.nlm.nih.gov/pubmed/25299267. PMID:25299267

12. Mukherjee S, Mukherjee B, Mukhopadhyay R, Naskar K, Sundar S, Dujardin JC, et al. Imipramine exploits histone deacetylase 11 to increase the IL-12/IL-10 ratio in macrophages infected with antimony-resistant *Leishmania donovani* and clears organ parasites in experimental infection. J Immunol. 2014 Oct 15;193(8):4083-94. doi: 10.4049/jimmunol.1400710. http://www.ncbi.nlm.nih.gov/pubmed/25217162. PMID:25217162

13. Rachidi N, Taly JF, Durieu E, Leclercq O, Aulner N, Prina E, et al. Pharmacological assessment defines *Leishmania donovani* casein kinase 1 as a drug target and reveals important functions in parasite viability and intracellular infection. Antimicrob Agents Chemother. 2014;58(3):1501-15. doi: 10.1128/AAC.02022-13. http://www.ncbi.nlm.nih.gov/pubmed/24366737. PMID:24366737

14. Knockaert M, Gray N, Damiens E, Chang YT, Grellier P, Grant K, et al. Intracellular targets of cyclin-dependent kinase inhibitors: identification by affinity chromatography using immobilised inhibitors. Chem Biol. 2000 Jun;7(6):411-22. http://www.ncbi.nlm.nih.gov/pubmed/10873834. PMID:10873834

15. Durieu E, Prina E, Leclercq O, Oumata N, Gaboriaud-Kolar N, Vougogiannopoulou K, et al. From Drug Screening to Target Deconvolution: a Target-Based Drug Discovery Pipeline Using *Leishmania* Casein Kinase 1 Isoform 2 To Identify Compounds with Antileishmanial Activity. Antimicrob Agents Chemother. 2016 May;60(5):2822-33. doi: 10.1128/AAC.00021-16. http://www.ncbi.nlm.nih.gov/pubmed/26902771. PMID:26902771
